# Assessment and simulation of a solid waste dumpsite impact on the surrounding water resources: A case study in Abu Zaabal, Egypt

**DOI:** 10.1016/j.heliyon.2021.e08421

**Published:** 2021-11-18

**Authors:** Mohamed E. El-Mathana, Nagwan G. Mostafa, Mona M. Galal, Abdelsalam Elawwad

**Affiliations:** Environmental Engineering Dept., Faculty of Engineering, Cairo University, El-Gamaa St., 12613 Giza, Egypt

**Keywords:** Landfills, Groundwater contamination, Simulation case study, Groundwater modeling

## Abstract

Indiscriminate dumping of solid wastes and the resulting groundwater contamination is a major issue, especially for developing countries. The main objective of this paper is to develop a groundwater mass transport model in order to study the effect of an open solid waste dumpsite on the water quality of water resources within the region around it. The harmful effects of indiscriminate solid wastes disposal by open dumping, which is still followed in many developing countries around the world, is highlighted. Abu Zaabal dumpsite; which is located in Qalyubiyah Governorate, Egypt; receives huge amounts of wastes daily causing leachate generation that percolates deep into the soil and polluting the shallow aquifer. The Groundwater Modeling System (GMS) software was used to model the groundwater flow and mass transport, using data collected from the site investigation and literature historical data available. Of the several contaminants measured in the site, six critical contaminants; namely Total Dissolved Solids (TDS), Lead, Boron, Nitrate, Manganese and Chemical Oxygen Demand (COD); were chosen to be modeled. The developed model was used to simulate the six contaminants using a transient-state model and concentration values for two different scenarios. Scenario-1 assumes that the dumpsite will be active until 2080, whereas Scenario-2 represents imminent closure of the dumpsite. The model results of each contaminant were calculated over 100-year interval, from 1980 until 2080, and the results of 2080 were presented. The results showed that the dumpsite had a major impact on the nearby water bodies, Abu Zaabal ponds and Belbais Drain. Moreover, the closure of the dumpsite showed that the maximum concentration of the majority of the considered contaminants was decreased by approximately 60–65%.

## 1 Introduction

Most of the developing countries rely on rivers and lakes as their main source of fresh water. However, with increasing population and demands, an alternative has to be established to meet such requirements. Similarly, Egypt is always reliant on the Nile River as the main source of fresh water and with increasing population, the demands are also increasing rapidly [1]. Therefore, the groundwater is always a practical alternative in order to meet the required needs. One of the major groundwater aquifers in Egypt lies under the Nile Delta region which is the Nile Delta aquifer. The Nile Delta aquifer is a huge renewable groundwater reservoir which is mainly recharged from the River Nile seepage, canals and drains leakage in addition to deep percolation from cultivated land [2]. However, this aquifer is at high potential risk of being polluted due to different contaminants generated from different sources such as exfiltration from sanitary sewers [3] and discharge of untreated sewage. As a result, the water quality of the groundwater has deteriorated over the past years [4]. One of the major sources of groundwater contamination is the dumpsites or landfills which are not correctly constructed as per engineering standards for the environment safety. Generally, landfills and dumpsites lead to environmental pollution, such as foul odor in the air, and leakage in form of leachate. Among all these, leachate leakage causes groundwater contamination and affects the groundwater quality as it consists of high concentrations of organic and inorganic compounds, heavy metals, toxic elements, and other harmful compounds. The surface water quality is rapidly deteriorating due to several pollution sources and since groundwater is the alternate source of fresh water, it is crucial to study the groundwater contamination transport and remediation as a key to help improve the groundwater quality. Therefore, one of the alternatives is using the groundwater as fresh water source and hence studying the groundwater contamination transport and remediation is a key to help improve the groundwater quality.

The simulation of mass transport problems is performed with a wide variety of specialized software such: (1) FeFLOW which uses finite element, and (2) Visual MODLFOW and Groundwater Modeling System (GMS) which uses finite-difference. The purpose of GMS which is applied in this study is to predict the dispersal of contaminants and its concentrations (C) knowing the initial conditions of groundwater flow direction, hydraulic head and the contaminants concentrations. GMS is considered one of the strongest and most sophisticated software that includes various packages such as the finite element groundwater model (FEMWATER), the multi-species reactive transport model (RT3D), the particle-tracking post-processing model (MODPATH), the modular three-dimensional transport model (MT3DMS), and the two-dimensional finite element model (SPEED2D) [5].

Several studies have been done previously in order to study the mass transport and the deterioration of the water quality due to contamination either from Landfills or any other [6, 7, 8]. In Egypt, many studies have been done on the Nile Delta region including South East where Abu Zaabal is located. These studies mainly focused on the concentrations of various contaminants and the prediction of future concentrations using the model.

Most of the studies which have been applied for mass transport modeling in the Nile Delta region usually covers a huge part, such as the East delta, the Western Delta or even the Central Delta region [9]. The research study done by El-Fakharany [9] was to construct a groundwater flow and mass transport model for the South-East Nile Delta region in Egypt. The main aim of the study was to use numerical modelling as a management tool to assess the groundwater quality of the Quaternary aquifer. The water resources in the study area are both surface water (freshwater canals and waste agricultural drains) and groundwater withdrawn from the Quaternary aquifer. The objective of the study was to predict the changes in levels of groundwater and quality concentrations using suggested schemes of water pumping with different abstraction rates for 30 years, which have been explored by Visual MODFLOW (version 3.0) and MT3D models. Another research study in Qalyubiyah Governorate, Egypt, performed by M.M. Galal [10] represents the inter-relation between surface water and groundwater and the impact of the surface water quality on the nearby shallow groundwater quality of the Nile Delta aquifer. A contamination transport model was generated using Visual Modlfow and MT3D and the results indicated that both surface and groundwater are affected by pollution from human and animal sources due to the absence of sewerage treatment systems and the disposal of domestic wastewater in the surrounding agricultural drains. It also revealed the role of clay cap thickness in hindering contaminants transport. Moreover, a study done by Chia-Huei [11] to construct a groundwater contaminant transport simulation of a decommissioned landfill at the city of Tainan in Taiwan. The main objective was to study the effect Wang-Tien landfill had on the study area's soil, groundwater and on the nearby Hsu-Hsian Creek which is known to fall in a region that has an unconfined aquifer. The model was done using Groundwater Modeling System (GMS) software utilizing both MODFLOW and MT3DMS tools. The landfill receives an accumulated amount of solid wastes around 773,970 m^3^ which started to dump the municipal solid waste since 1992 and was abandoned in 2002. Nevertheless, according to a study performed by T. Khayyun [12] the effective porosity is a major parameter that is highly sensitive during the contaminant transport process. The study was performed in Iraq and focuses on the simulation of groundwater flow and contaminant transport of radioactive Cobalt-60 (Co-60) by advection and dispersion processes using MODFLOW and MT3DMS. The leakage of Cobalt-60 into the groundwater was due to the nuclear incident resulted from operating a Metal Testing Laboratory Nuclear Facility. The period of simulation started in 2016 and the model showed that Co-60 concentrations after 5 and 10 years were 32.34–34.44 μg/m^3^ and 34.86–37.31 μg/m^3^ respectively. A remedial process consisting of three pumping wells which were fully penetrating the aquifer were used to limit and control Co-60 movement.

The main objective of this research is to study the impact of Abu Zaabal dumpsite on the nearby water resources using GMS modeling software developed by Aquaveo Engineering Services company. The dumpsite receives a huge amount of solid waste around 3.5 tons/day [13]. The solid waste produces leachate which percolates into the soil to the groundwater, and then it spreads by groundwater flow, which is known as mass (contaminant) transport. The aim of this research is to study the impact of contaminated leachate on the water quality of the nearby water resources under two main scenarios; continuous operation and closure of the dumpsite. The numerical modeling in this study is performed using GMS software.

## 2 Methodology, methods and materials

### 2.1 Study process

**Figure 1:**
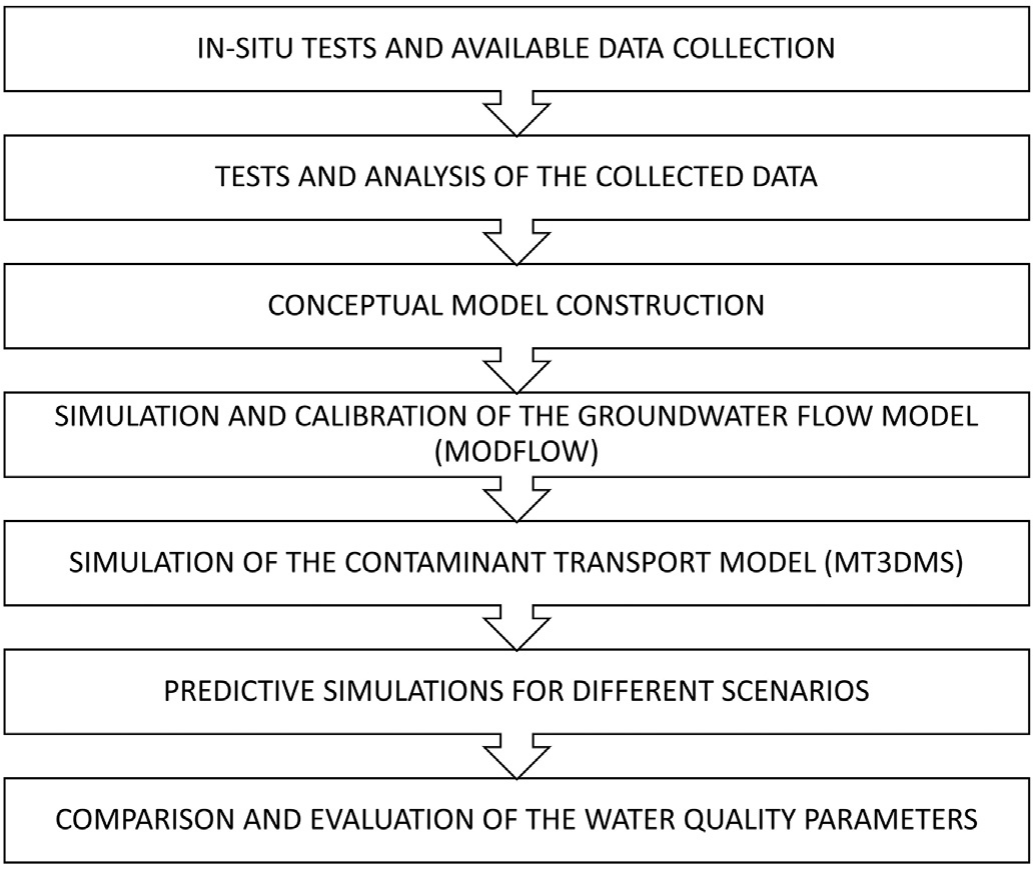
Flowchart of the study process.

The following steps shown in Figure 1 are the steps followed in this research. This includes the construction of the numerical model which combines both Groundwater Flow and Mass Transport in order to analyze and study the water quality condition within the study area.

### 2.2 Governing equations

#### 2.2.1 Groundwater flow equation

MODFLOW is an international public domain and an industry-standard groundwater modeling [5] developed and enhanced by United States Geological Survey (USGC) scientific agency. It was used for groundwater conditions evaluation and constructing a three-dimensional mathematical model of the aquifer in this study.

A small representative elemental volume (REV) is often used to derive the groundwater flow equation and the properties of the medium are often assumed to be effectively constant, to derive the groundwater flow equation [14]. The mathematical model for groundwater flow consists of two equations:

⁃ Equation of motion, Darcy equation.
⁃ Equation of continuity.

The combination of the aforementioned equations forms the general groundwater flow equation, Eq. (1):

(1) $$\frac{\partial }{\partial x}\left(T_{x}\frac{\partial h}{\partial x}\right)+\frac{\partial }{\partial y}\left(T_{y}\frac{\partial h}{\partial y}\right)+W=S\frac{\partial h}{\partial t}$$

where:

⁃ *T*_*x*_ and *T*_*y*_ are respectively the transmissivity in x and y directions $\left(T_{x}=b.K_{x}\;\text{and ​}T_{y}=b.K_{y}\right)$, [L^2^/T];
⁃ *h* is the piezometric head, [L];
⁃ *W* is the volumetric flux per unit area representing sinks/sources, [L/T];
⁃ *S* is the storativity or storage coefficient $\left(S=S_{y}+b.S_{s}\right)$, [-];
⁃ *S*_*y*_ is the specific yield, [-];
⁃ *S*_*s*_ is the specific storage, [1/L];
⁃ *b* is the saturated thickness, [L].

#### 2.2.2 Groundwater mass transport

The partial differential equation which describes the groundwater mass (contaminant) flow transport of a species (k) through a porous medium in three-dimensional transient flow is shown in Eq. (2) [15]:

(2) $$\frac{\partial \left(\theta C^{K}\right)}{\partial t}=\;\frac{\partial }{\partial x_{i}}\left(\theta D_{ij}\frac{\partial C^{k}}{\partial x_{j}}\right)-\frac{\partial }{\partial x_{i}}\left(\theta v_{i}C^{k}\right)+q_{s}C_{s}^{k}+\sum R_{n}$$

where:

⁃ θ: subsurface medium porosity, [-];
⁃ $C^{k}$: dissolved concentration of species k, [ML^−3^];
⁃ t time, [T]
⁃ $x_{i}$: distance along respective Cartesian coordinate axis, [L];
⁃ $D_{ij}$: hydrodynamic dispersion coefficient tensor, [L^2^T^−1^];
⁃ $v_{i}$: linear pore water velocity or seepage, and it is related to Darcy flux or the specific discharge through and can be calculated as $v_{i}=\frac{q_{i}}{\theta }$ , [LT^−1^];
⁃ $q_{s}$: volumetric flow rate per unit volume of aquifer representing fluid sources (positive) and sinks (negative), [T^−1^];
⁃ $C_{s}^{k}$: sink flux for species k or concentration of the source, [ML^−3^]; and
⁃ $\sum R_{n}$: chemical reaction term, [ML^−3^T^−1^].

### 2.3 Site description

**Figure 2:**
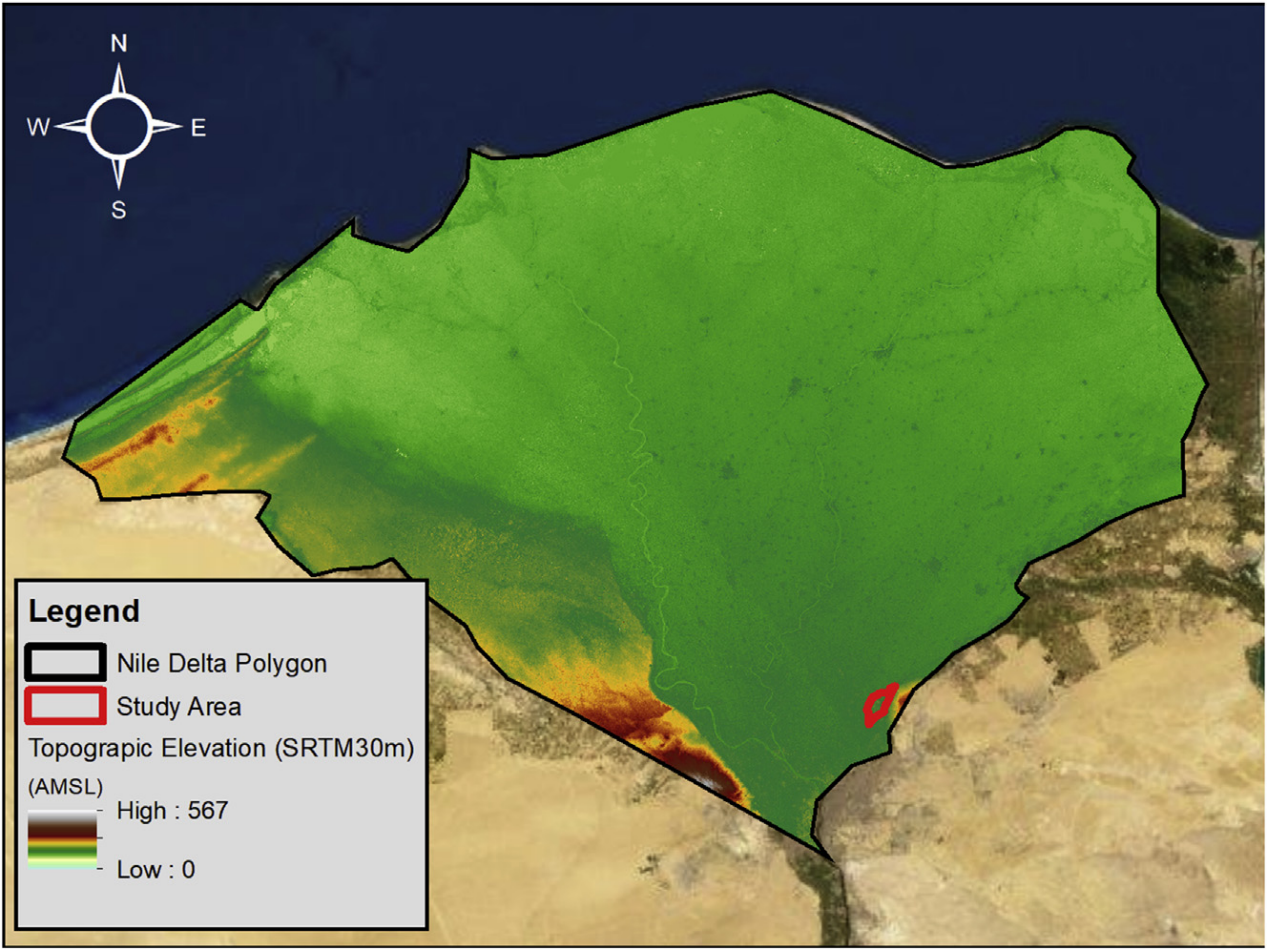
Regional topographic elevations along with site location.

**Figure 3:**
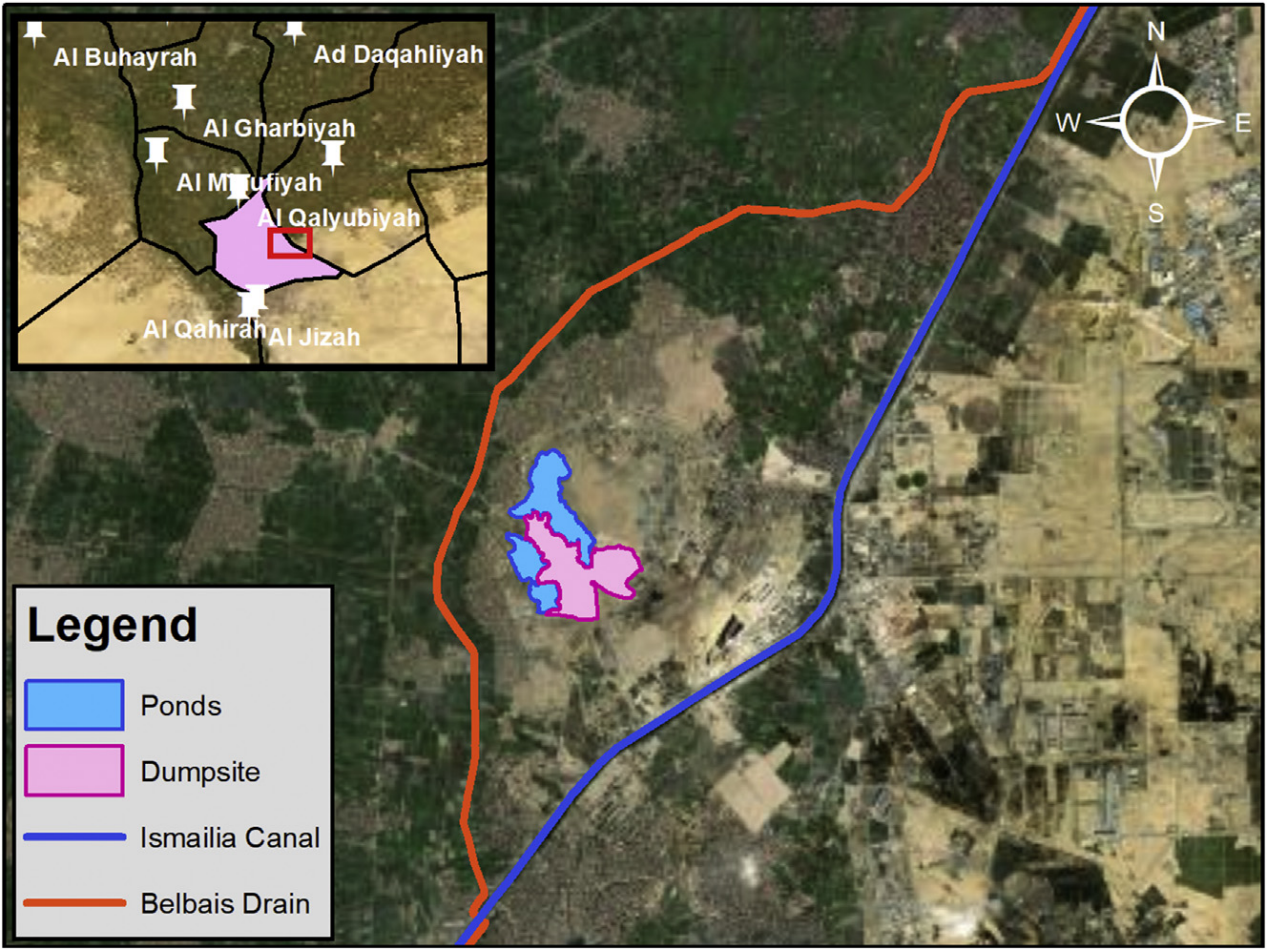
Local site location.

The study area which encompasses Abu Zaabal Dumpsite is located in Qalyubiyah Governorate, South-East of the Nile Delta region as shown in Figures 2 and 3. Abu Zaabal Dumpsite is in a very sensitive location being bordered by Belbais Drain from the west, Ismailia Canal branch from the East and having many agricultural fields in the region. Taking into consideration the huge quantity of waste (8.4 million m^3^) that are dumped there, makes it a potential contamination threat from domestic and industrial sources. The dumpsite has been operating for approximately 30–40 years, which upon it, the model starting date has been decided [16].

The area surrounding the dumpsite was used in the past as a Quarry known as “Abu Zaabal Quarries” where basaltic rock sheets were extracted. The land use in the study area includes residential places, industries, treatment plants, solid waste dumpsites, agricultural lands, and several water bodies such as ponds, drains and canals. The main usage of the canals and drains is for Irrigation purposes and according to the people of Abu Zaabal, the ponds are not used except for a limited part where fish farm that can tolerate high salinity was developed [17].

**Figure 4:**
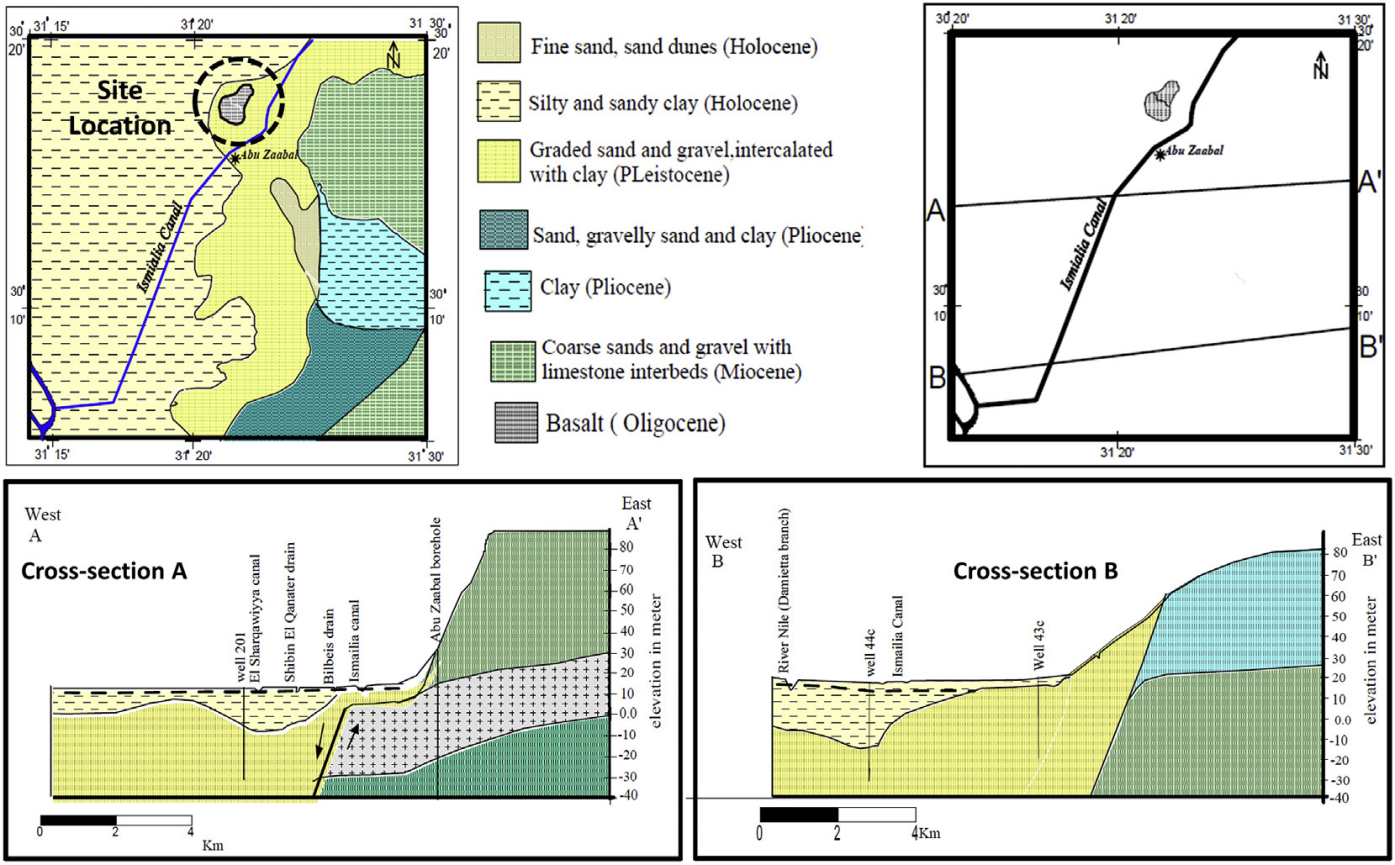
Regional Geological Map and cross-sections near to the study [18].

The geology of the study area mainly consists of quaternary sediments which are graded sand and gravel intercalated with clay which refers to the Pleistocene age as well as basaltic rocks belonging to upper Oligocene age. According to other several sources, it is also stated that the presence of Sandstone goes back to either the Miocene age or the Oligocene age [18]. Figure 4 represents the regional geological map of the study area and a key map for two cross-sections. The two cross-sections ‘A’ and ‘B’ cutting through the subsurface which is nearby the study area indicate the extent of the basalt and different other geological formations. The hydrogeology of the study area is part of the East Nile Delta which is quite complicated due to the existence of Abu Zaabal quarries. The main aquifer within the study area is the Quaternary Aquifer which falls under the Nile Delta Aquifer system. The Quaternary aquifer is divided into two hydrogeological units; the Holocene aquitard which is moderately productive and the Pleistocene aquifer which is highly productive. The Oligocene aquifer which represents Abu Zaabal ponds is hydraulically connected to the Quaternary aquifer through faults, fractures and joints.

The main water resources within the vicinity of the study area are Ismailia Canal, Belbais Drain and Abu Zaabal Ponds. Generally, the surface water levels of canals and drains increase during summer period and decrease during winter period in response to changes in the irrigation water quantities. Liquid wastes are discharged into the drains and on the land surface. Therefore, the polluted water infiltrates contaminating the Quaternary (Pleistocene) aquifer. Abu Zaabal ponds are located in the north of Qalubiyah Governorate 30 km of Cairo. These Ponds are closed inland basins and they are man-made, formed as a result of rocks mining in this area. The water inside the ponds mainly came from the seepage water from the surface and Ismailia Canal [19]. Originally, they were four ponds but the smallest pond has been buried under the solid waste being dumped there for years. The ponds cover an area of approximately 600,000 m^2^.

**Figure 5:**
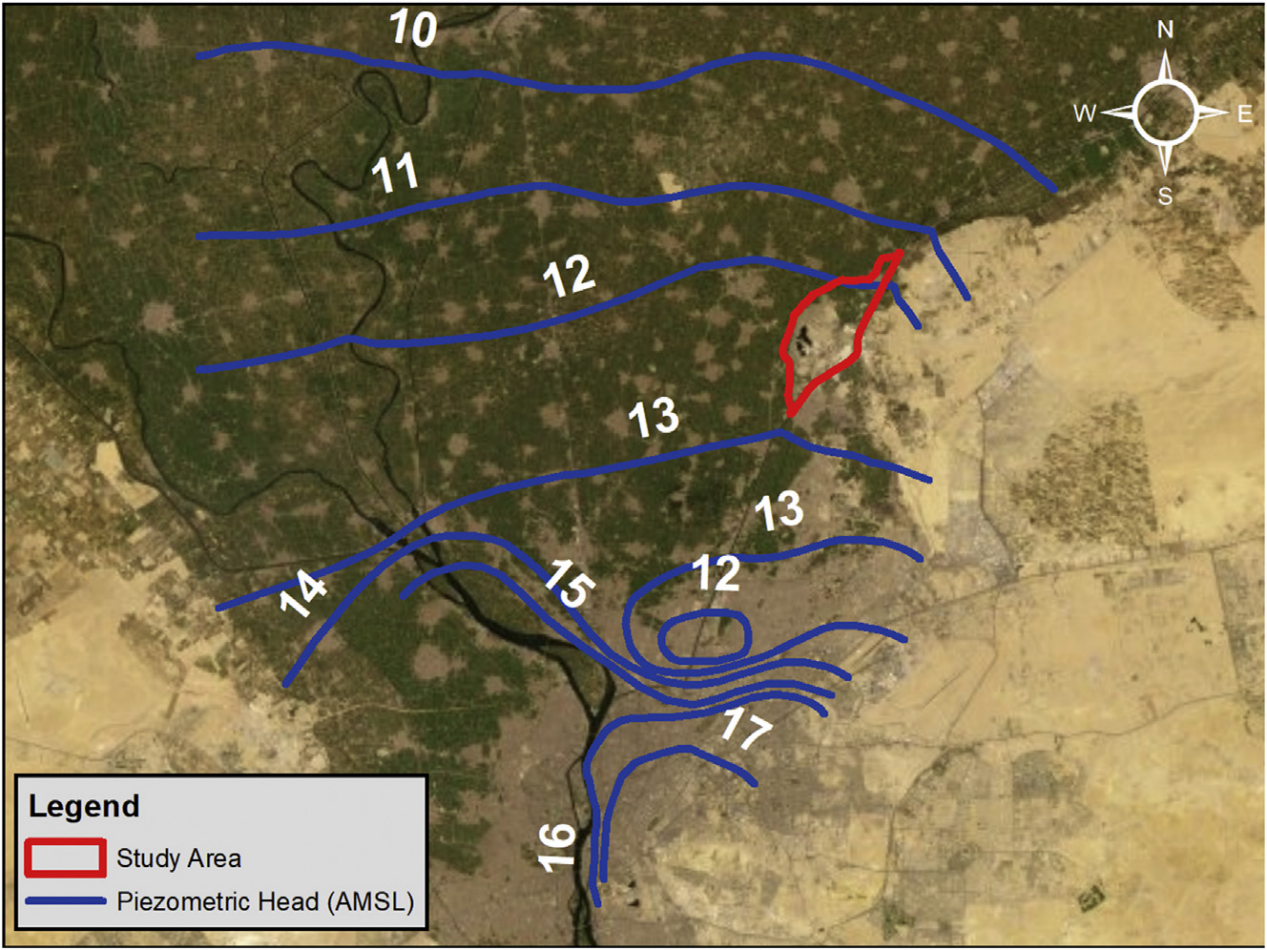
Quaternary aquifer Groundwater level contours for Nile Delta.

The regional groundwater levels of the Quaternary aquifer in the Nile Delta were measured mainly using existing wells at different locations. The site falls in the East of the delta where the groundwater level according to Hydrogeological map of Egypt for the Nile Delta ranges between 10-13 AMSL (Above Mean Sea Level) [20] as shown in Figure 5. The groundwater flow direction within the region of the study area is from the East to the West; i.e. from Ismailia Canal to the Belbais Drain.

### 2.4 Field work and laboratory analysis

**Figure 6:**
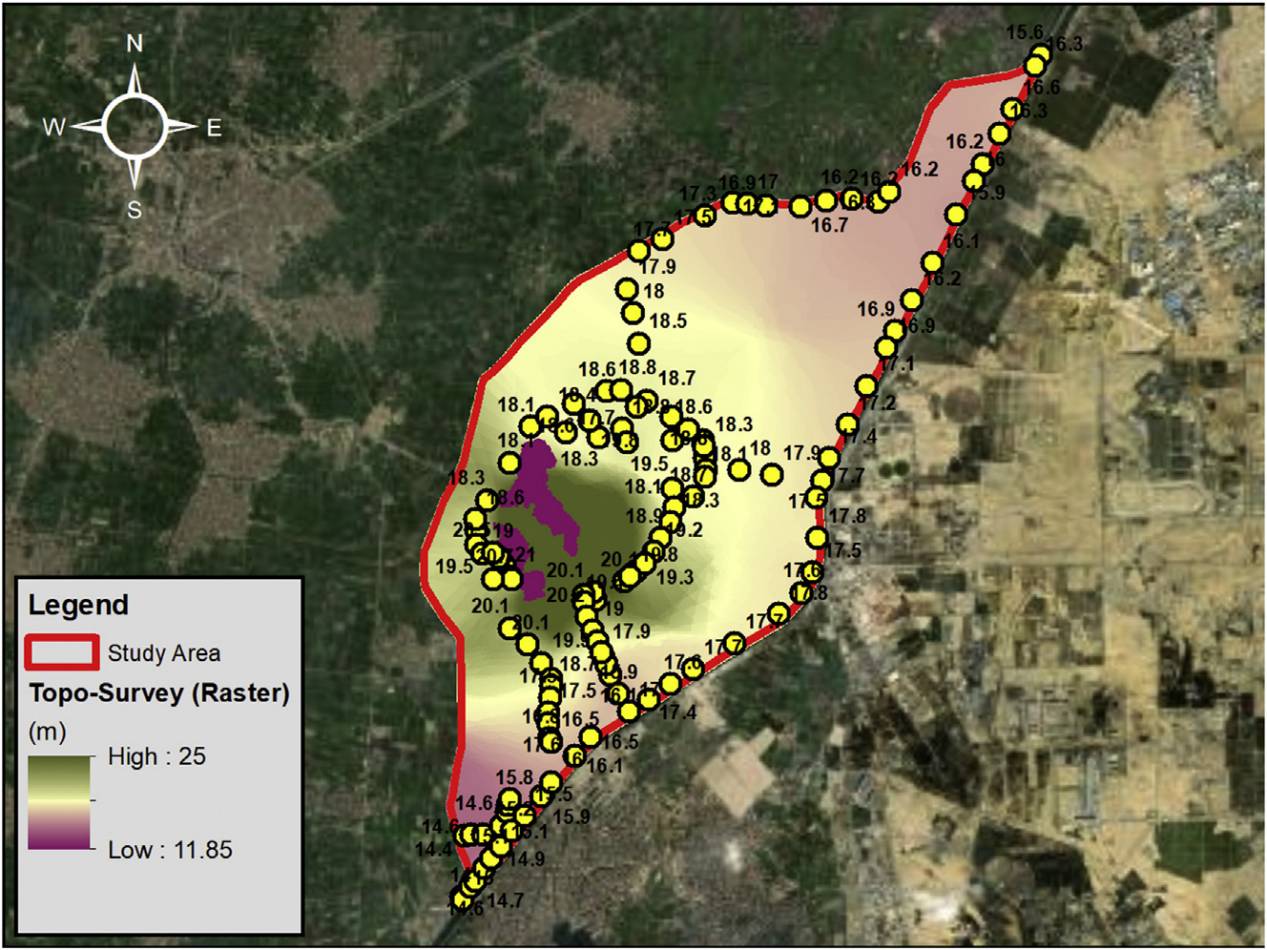
Topographic Survey using GPS for the study area.

Besides the information collected from previous studies, historical data and literature review, an extensive site investigation was carried out in July–August 2019. Firstly, The Topographic Elevations of the site has been obtained using Global Positioning System (GPS) by a Surveying Team under supervision of Department of Geomatics, Faculty of Engineering, Cairo University as part of the site investigation performed. The spot levels were converted into a Digital Elevation Model (DEM) in order to cover the whole study area as shown in Figure 6. However, outside the border of the study limit, the Shuttle Radar Topography Mission (SRTM) a free source was used in order to generate a combined source for the ground elevations to be used in the model.

**Figure 7:**
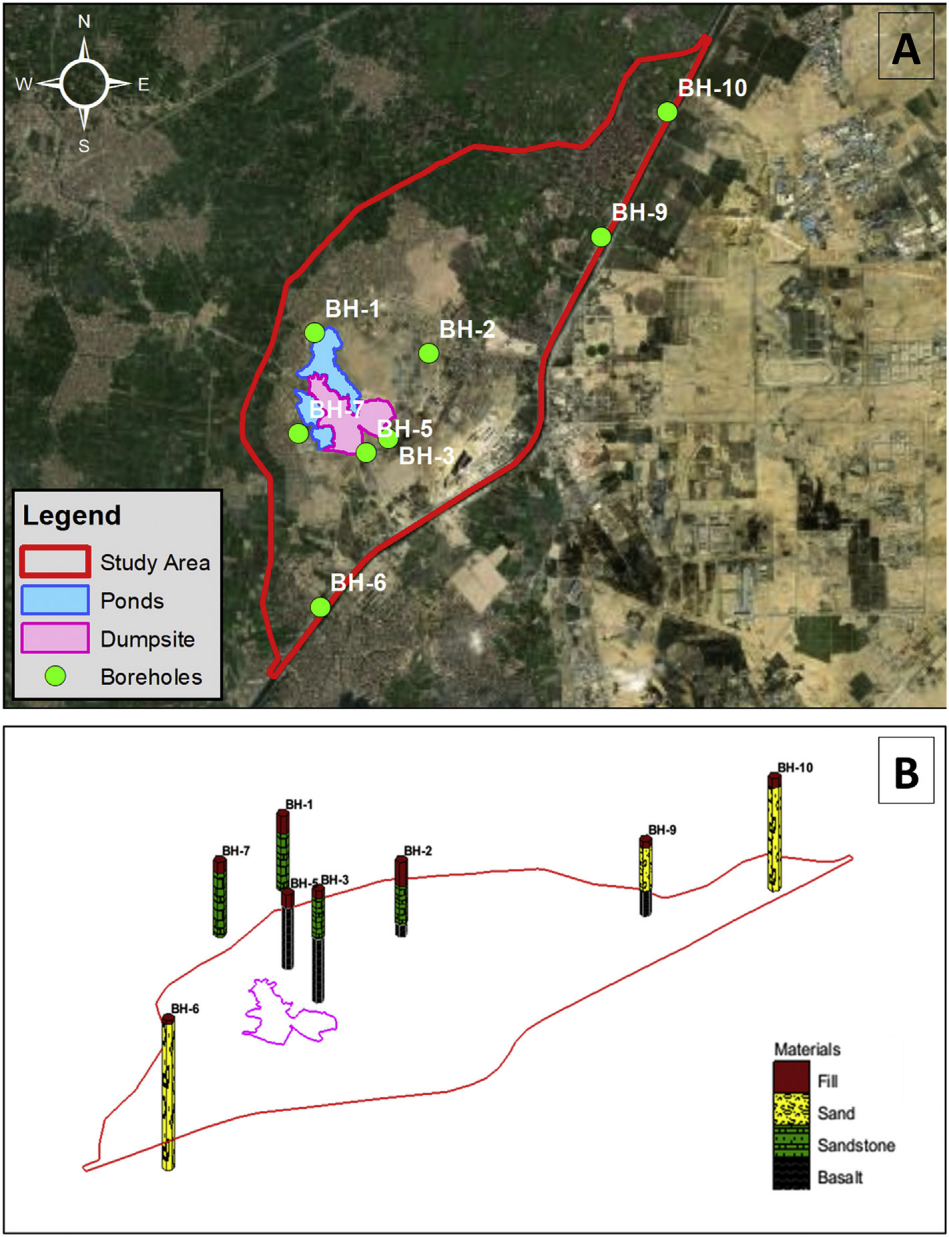
The boreholes: (A) locations and (B) 3D view showing encountered lithology.

Secondly, the site investigation performed by Soil Mechanics and Foundations Research Laboratory, Faculty of Engineering, Cairo University included drilling of 8 boreholes within the boundaries of the study area in order to have a more detailed and specific description of the type of lithology present locally. The location of the boreholes is shown in Figure 7. A 3-Dimensional view was also generated using Groundwater Modeling System (GMS) lithological modeling module (See Figure 7). The main lithological formations found were fill, sand, sandstone and basalt. Based on the Rock Quality Designation (RQD) which indicates the fracture or jointing degree in a rock mass in percentage; most of the rocks were completely weathered having an RQD of less than 25%.

**Figure 8:**
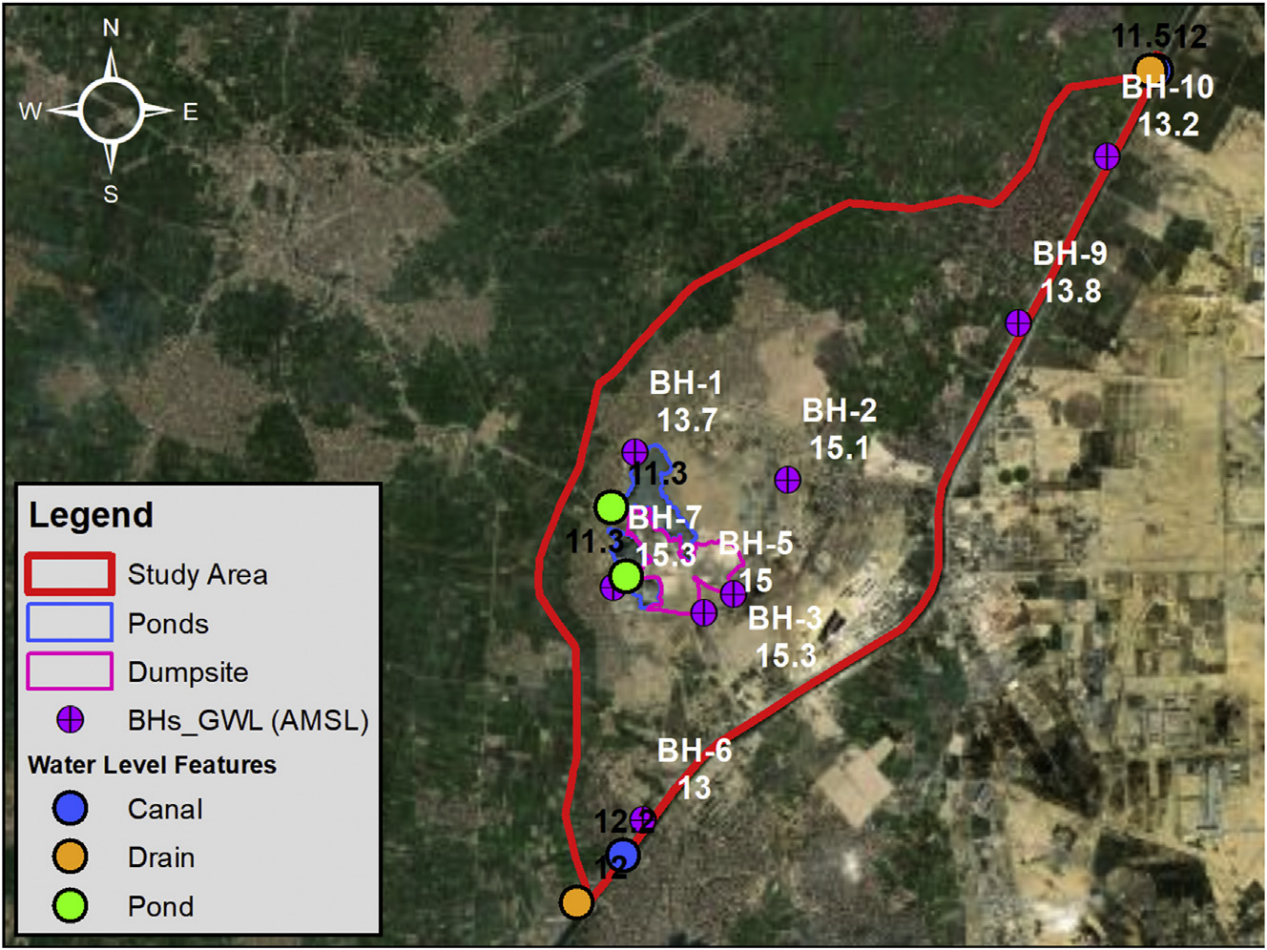
Groundwater level and surface water level at different water bodies.

Thirdly, the water levels in Ismailia Canal, Belbais Drain and Abu Zaabal ponds were measured with the help of the Surveying Team at different locations each as shown in Figure 8. The water level was measured upstream and downstream of the study area for Ismailia Canal and Belbais Drain whereas the two water level measurements were taken also for the ponds at accessible locations. The water level in the canal ranges from 12.2m AMSL to 12.0m AMSL and at the drain from 12.0m AMSL to 11.5m AMSL. Groundwater levels were also measured in the eight (8) drilled boreholes as shown in Table 1. This was performed in order to have current groundwater level readings to be able to have a better understanding of the changes in the piezometric head and the factors affecting it. Figure 8 shows the locations of the boreholes including the groundwater level readings measured at the boreholes.

**Table 1:** Site Investigation groundwater level and surface water level measurements.

Location	Ground Elevation (m AMSL)	Groundwater Depth (m)	Groundwater Level (m AMSL)	Water Level (m AMSL)
BH-1	18.6	4.8	13.7	-
BH-2	18.2	3.1	15.1	-
BH-3	20.6	5.3	15.3	-
BH-5	20.5	5.5	15.0	-
BH-6	16.0	3.0	13.0	-
BH-7	20.8	5.5	15.3	-
BH-9	16.6	2.8	13.8	-
BH-10	16.2	3.0	13.2	-
Ismailia Canal Upstream	-	-	-	12.2
Ismailia Canal Downstream	-	-	-	12.0
Belbais Drain Upstream	-	-	-	12.0
Belbais Drain Downstream	-	-	-	11.5
Pond (1)	-	-	-	11.3
Pond (2)	-	-	-	11.3

**Figure 9:**
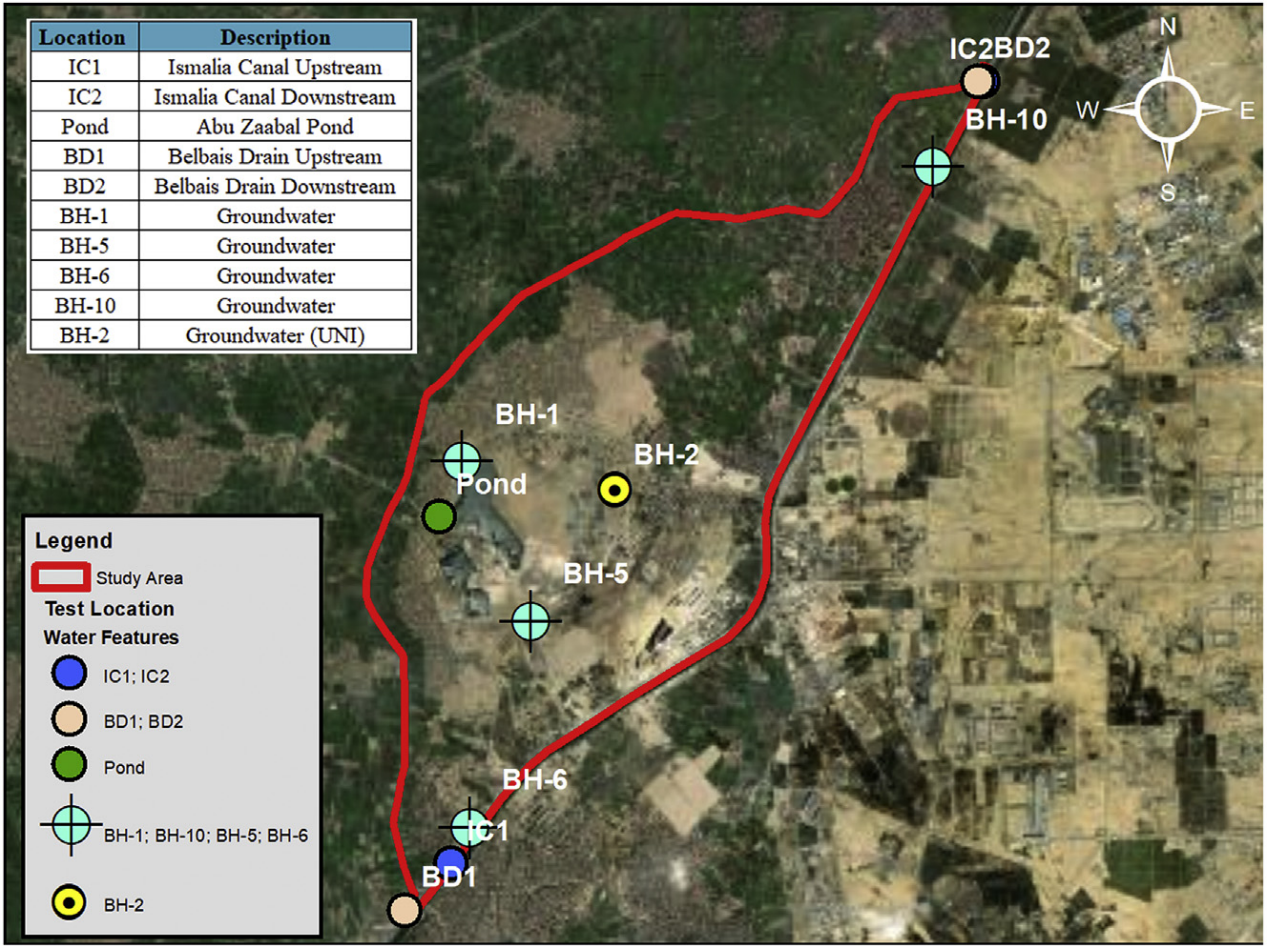
Locations of test samples collected from surface water and groundwater.

Fourthly, water samples were taken from surface water (Ismailia Canal, Belbais Drain, and Abu Zaabal Ponds) and Groundwater from the drilled boreholes under the supervision and guidelines of the Agricultural Research Center (ASR). Chemical and biological analyses were performed on the samples that were tested according to the standard method for the examination of water and wastewater [21]. The locations of the tests are shown in Figure 9.

Finally, the composition and quantities of the solid waste dumped at Abu Zaabal were derived from the Annual Report of Solid Waste Management [13] for the city of Qalubiyah. The main dumpsite which receives most of the solid waste is Abu Zaabal. The solid waste production ranges from 1.0 kg/person/day in urban areas to 0.7 kg/person/day in rural areas. The approximate solid waste density is 300 kg/m^3^ and the moisture content is 30%, which is a reasonable percentage since most of the waste in Abu Zaabal dumpsite are organic materials. According to the Ministry of Local Development, the components of solid waste in Qalyubiah are divided into 20% organics, 11.5% paper and cardboard, 15% plastics, 10% glass, 5% metals and 38.5% other wastes. Limited data is available regarding Abu Zaabal dumpsite and due to site conditions, it was difficult to estimate the leachate content. However, according to some studies, it can be assumed that the amount of leachate generation is around 3 m^3^/day/ha [7, 22, 23]. Since the landfill areas is approximately 630,000 m^2^ (63 ha), therefore, the leakage from the dumpsite can be estimated to be 189 m^3^/day. Additionally, dumpsites and landfill are also a potential threat to the subsurface groundwater in case of any leakage that occurs. A research study was done by M. Abd El-Salam [22] at Borg Al-Arab Landfill in Alexandria which mainly focused on examining the effect of the leachate generated by the solid waste in the landfill on the environment and the surrounding groundwater. Several physio-chemical parameters were tested and analyzed from samples taken from the groundwater and the leachate pond (Table 2) where it was disposed of. The results of this study gave an overview of the values of each parameter that were used as guidance in developing the mass transport model in this research.

**Table 2:** Physical and Chemical analyses of leachate and groundwater samples collected from Borg Al-Arab landfill in Alexandria [22],

Parameter	Units	Leachate Sample (Min-Max)	Mean ± SD	Groundwater Sample (Min-Max)	Mean ± SD
Tota1 Dissolved Solids	mg/l	24,954–30,482	27,452 ± 605	2855 - 16,276	9308 ± 75
Lead	mg/l	0.008–0.025	0.019 ± 0.004	0.002–0.009	0.003 ± 0.0005
Manganese	mg/l	0.26–1.39	0.839 ± 0.165	0.039–0.673	0.307 ± 0.2
Nitrate	mg/l	0.36–2.90	1.4 ± 0.2	0.05–0.36	0.19 ± 0.045
Chemical Oxygen Demand	mg/l	12.85–16,350	15,629 ± 206	45–190	74 ± 3
Biochemical Oxygen Demand	mg/l	9620 - 11,700	10,824 ± 95	16–95	52 ± 1

### 2.5 Numerical modeling

#### 2.5.1 Groundwater flow model

The groundwater flow model for the study area in Abu Zaabal is constructed using Groundwater Modeling System (GMS) which utilizes MODFLOW as the main module. The main challenge of the groundwater flow model was the simulation of Abu Zaabal ponds along with the other water bodies within the study limit. The first model was developed using Lake (LAK) Package to simulate the ponds [24]. However, because the mass transport model (MT3DMS) does not support the LAK package [25], in the later stages it was replaced by River (RIV) package using the same characteristics. The LAK package helped determine the Leakance which is defined as the conductance in the RIV package that shows the hydraulic relation between the ponds and the aquifer.

**Figure 10:**
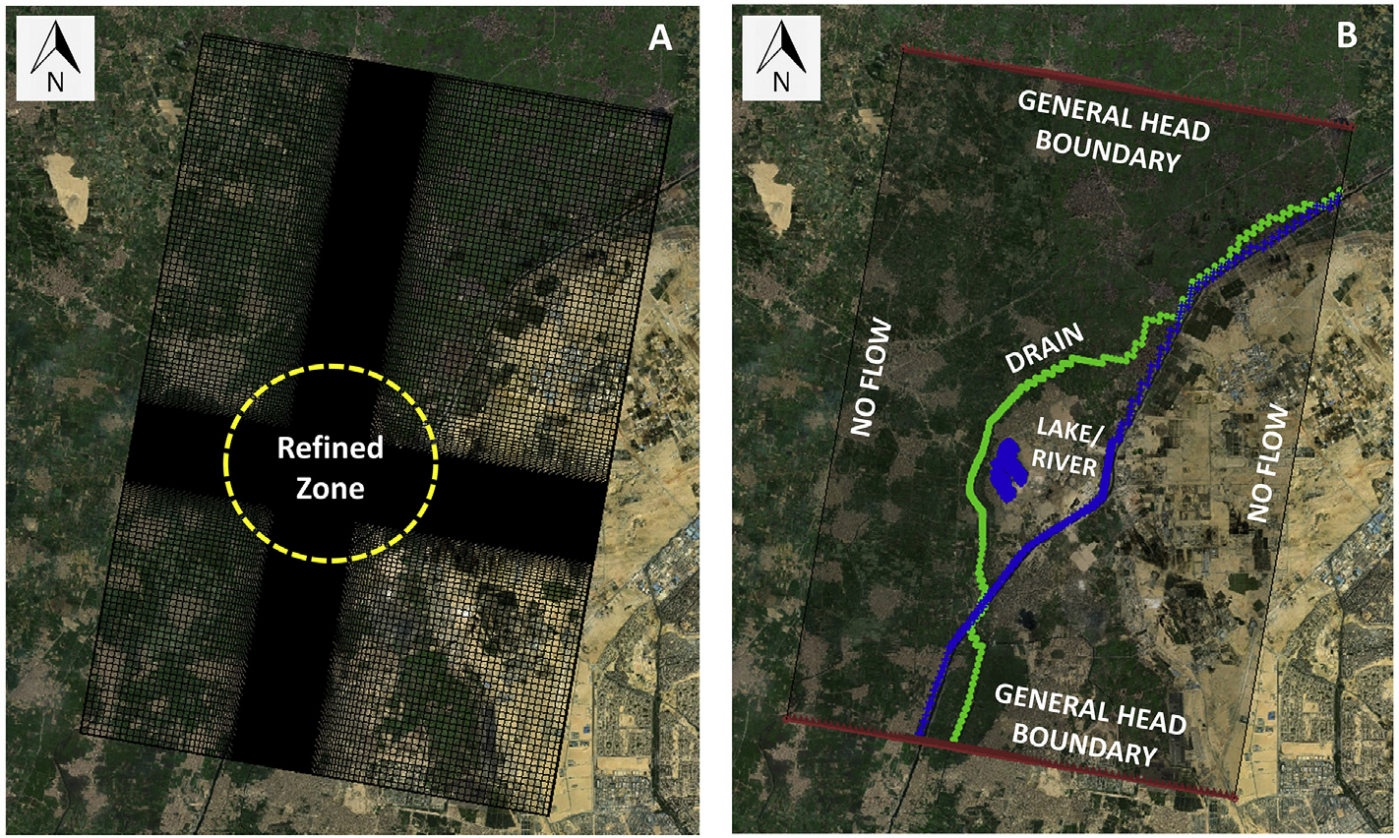
A) Model Grid, B) Model boundary conditions, sources and sink.

A single layer model (14Km∗21Km) has been constructed. The grid was oriented with the expected flow direction. The grid is refined at the site and the cell size is variable due to the refinement near the site as shown in Figure 10. The minimum cell size is 20m∗20m (around the Dumpsite) and the maximum cell size reaches 200m∗200m. The boundaries (General Head Boundary and No Flow Boundary) were taken far away from the study area to minimize the effect of boundary condition. The Ismailia Canal being a huge stretch was simulated as River (RIV package), the Belbais Drain as a Drain (DRN package) and the ponds as River (RIV package). The Top Elevation of the model was assigned by the generated combined SRTM and Topographic Survey, and the Bottom Elevation was set to -30.00m AMSL. The conductance of the ponds (RIV) was assigned the value obtained from the Leakance during the initial run using LAK package. The value was 0.0005 (m^2^/day)/m^2^. This value is verified by having the water level in the pond lower than the water level outside.

**Figure 11:**
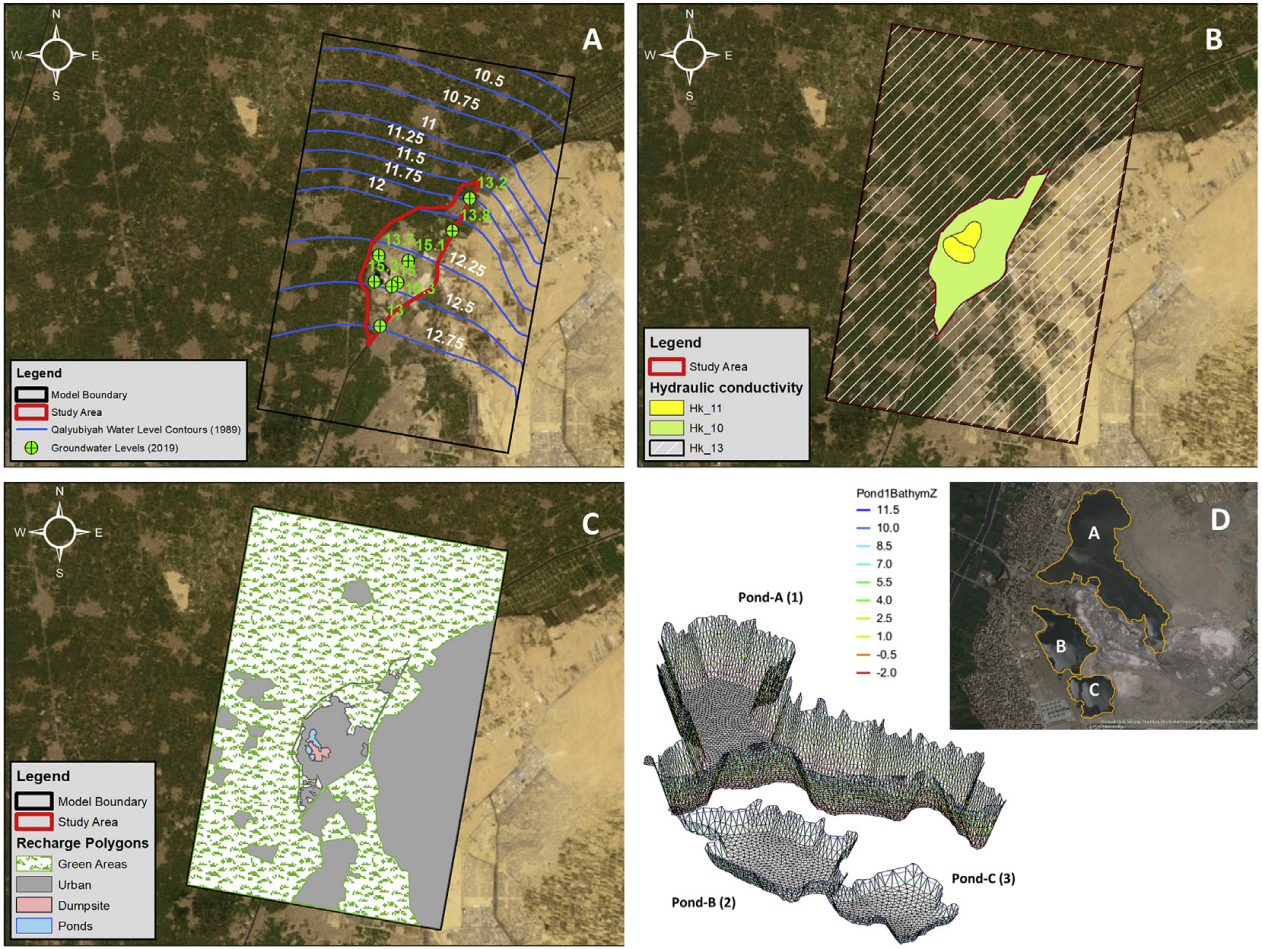
A) Groundwater levels used in model calibration, B) Model Hk zones, C) Model Recharge zones, D) Ponds bathymetry created using TIN.

The head of the General Head Boundary (GHB) was assigned using the historical groundwater level contours which is 13 MSL in the south and 10.5 MSL in the north. The Drain (Belbais) top level is 12 AMSL at the upstream and 11.5 AMSL downstream. The River (Ismailia Canal) top level is 12.5 AMSL upstream and 12 AMSL downstream. The pond water level which is assigned as a lake has a water level of 11.85 AMSL. Moreover, the LAK package also requires the bathymetry of the ponds as input. Since no such data is available, it has been created using Geographic Information System (ARCGIS) software using the gathered site survey and the literature information available related to the ponds depths. The Triangular Irregular Networks (TIN) method has been used to generate the approximate bathymetry of the ponds as shown in Figure 11.

The features within the model boundary such as agricultural lands, urbanized areas, ponds, canals, drains and dumpsite have been digitized. Recharge has been assigned to the model according to the land use. Irrigation lands (Green Areas), Urbanized Areas, Ponds and Dumpsite were all given recharge values. Green Areas and the Dumpsite were given the same Parameter Flag, whereas the Urbanized Areas were given a different Parameter Flag which represents leakage from infrastructure and wet utilities. Evapotranspiration has been assigned to the whole model with a value of 0.0005 m/d per unit area and an extinction depth of 0.5 m [26].

Hydraulic conductivity was assigned to the whole model by three (3) different zones as shown in Figure 11. A zone representing the Basalt outcrop (Hk_11) where the hydraulic conductivity is low (Miocene aquifer), a zone representing a relatively high hydraulic conductivity (Hk_10, Quaternary; Pleistocene aquifer) and the third zone represent the area outside the study area (Hk_13). Furthermore, specific yield (Sy) and specific storage (Ss) of the aquifer were also included in the model.

The calibration of the model was based on groundwater level data available from the Hydrogeological map of Egypt-Nile Delta [20] and the readings were taken during the site investigation as shown in Figure 11. The model was divided into two parts; a steady-state part from 1980 till 1990 where the groundwater level contours were used and the transient part from 1990 till 2020 where the recent site investigation groundwater levels are used. The list of parameters that were estimated during the calibration and process and their initial assigned values are shown in section-3.

#### 2.5.2 Contaminant transport model

In integration with MODFLOW using GMS, the Modular 3-Dimensional Transport model Multi Species (MT3DMS) was used to generate the mass transport model. The mass transport model has been set up based on the following:

- Recharge concentration from the Dumpsite (Leachate) for different pollutants
- Recharge concentration into/from the ponds to the groundwater
- Assigning specific concentration to Ismailia Canal
- Assigning starting concentration to the Study Area and Ponds

There were a variety of water quality parameters tested in the study area during the site investigation (2019) and from historical data. However, the major contaminants that will be simulated in the model are six parameters, namely: Total Dissolved Solids (TDS), Chemical Oxygen Demand (COD), Nitrates, Manganese, Lead and Boron. These contaminants have been chosen due to their high concentration levels or the potential high risk that could cause concern in the future.

The mass transport model is simulated based on two scenarios. This includes simulating the mentioned contaminants from 1980 (approximately the year where the dumpsite started) until 2020, which will be considered as the current condition. The two projected scenarios are:

➢ Scenario-1: Assuming Continuous Operation of the Dumpsite
➢ Scenario-2: Assuming Closure of the Dumpsite at year 2020

The mass transport model (MT3DMS) operates using three main packages, The Advection package default solver was used which is the Third order TVD scheme (Ultimate). Longitudinal Dispersity (α) was 20 [27] the ratio of vertical transverse dispersivity to longitudinal dispersivity (TRVT) was assumed to be 0.01m. and the ratio of horizontal transverse dispersivity to longitudinal dispersivity (TRPT) was 0.5 m.

The chemical reaction package is used in the model to simulate the effect of retardation on the groundwater pollutant. This retardation causes a slowdown and concentration decrease in the migration of the contaminant by processes such as sorption (adsorption, absorption) and biodegradation. This package is used only for COD. Few parameters needed to be identified in the package based on the aquifer and contaminant properties. The following assumptions were made:

1. Kinetic rate of reaction: First Order
2. Bulk density (kg/m^3^) of the subsurface medium or porous medium (aquifer); according to literature and previous studies it ranges from 1600-2200 kg/m^3^ depending whether it is sand, sandstone or basalt; 2000 kg/m^3^ was adopted as average [27].
3. Rate constant (dissolved) λ = 0.0004
4. Rate constant (sorbed) λ = 0.0004

**Figure 12:**
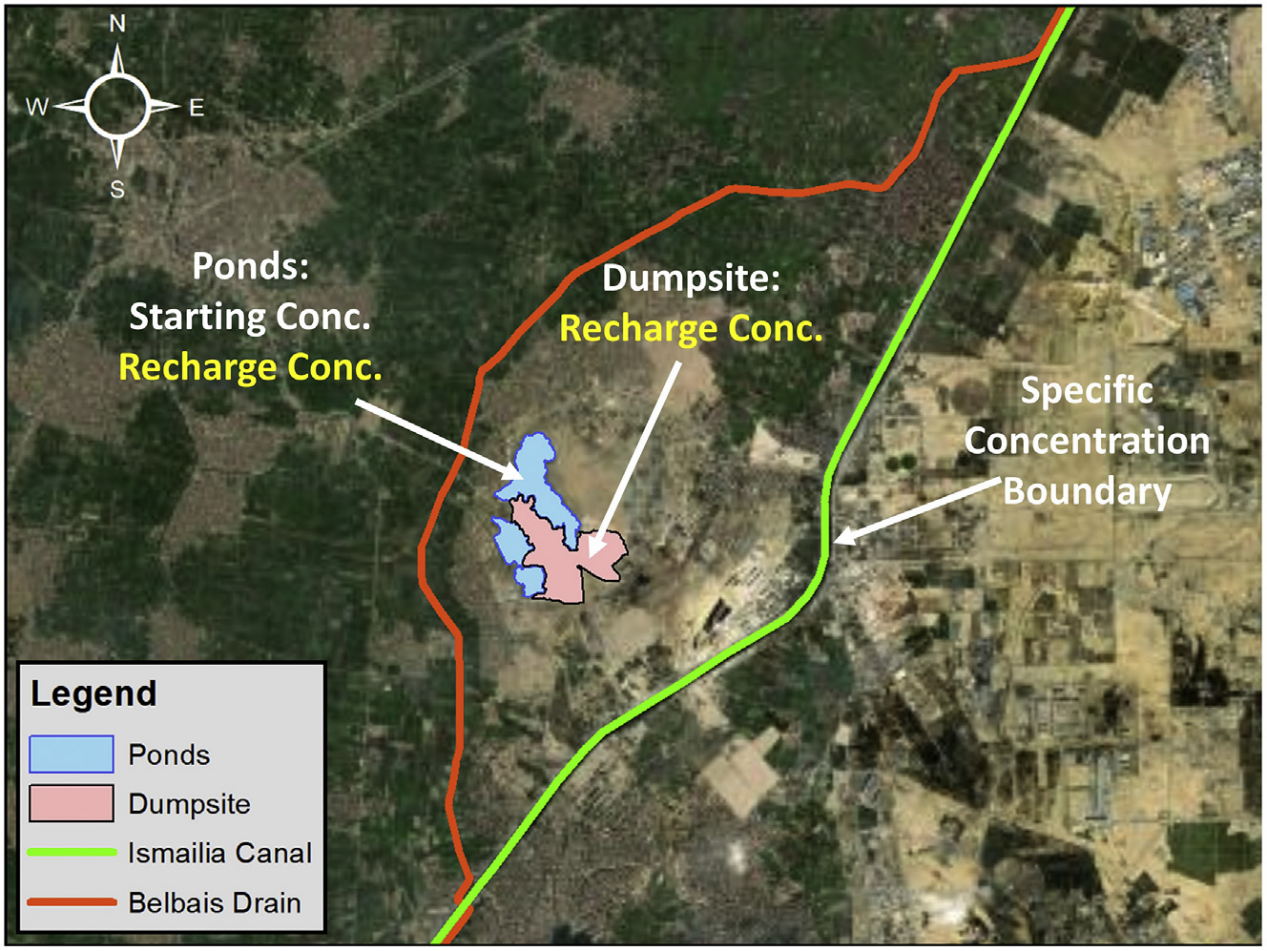
Location of recharge concentration, starting concentration and concentration boundaries assigned to the model.

Table 3 and Figure 12 show the different locations and initial input parameter values of the recharge concentration, initial (starting) concentration, and concentration boundary conditions assigned to the model.

**Table 3:** Initial concentration, Recharge concentration values for different contaminants.

Contaminant	Starting Concentration (Study Area)	Starting Concentration (Pond)	Ismailia Canal	Recharge Concentration (Dumpsite)
TDS	200	500	300	30,000
COD	2	2	10	10,000
Nitrate	0.5	0.5	1.5	2
Manganese	0.2	0.5	0.25	1
Boron	0	0	0.28	2
Lead	0	0	0.065	0.02

## 3 Results and discussion

### 3.1 Groundwater flow model

Several trials were performed to conclude the groundwater flow model calibration. After several iterations and improvements, the following results were obtained with a calculated sum of squared weighted residual error of 68.5, and a good fitting was achieved. The optimized results of the calibration are shown in Table 4.

**Table 4:** Initial and calibrated values for different parameters.

Parameter Flag	Description	Initial Values	Calibrated Values	Units
HK_10	Hydraulic conductivity (High)	15	10	m/day
HK_11	Hydraulic conductivity (Low)	2	0.95	m/day
HK_13	Hydraulic conductivity (outside study area)	5	100	m/day
GHB_8	General Head Boundary Conductance	10	6.15	(m^2^/day)/m
GHB_7	General Head Boundary Conductance	10	100	(m^2^/day)/m
SS_60	Specific Storage	1.00E-05	1.00E-06	1/m
SY_70	Specific Yield	0.2	0.1	
RCH_20	Recharge (Irrigation Lands)	1.00E-05	2.66E-04	m/day
RCH_21	Recharge (Irrigation Lands)	1.00E-05	6.07E-04	m/day
RCH_30	Recharge (Urban Leakage)	1.00E-06	2.00E-08	m/day
RCH_31	Recharge (Urban Leakage)	1.00E-06	1.02E-04	m/day
RIV_40	River Conductance (Ismailia Canal)	0.01	0.87	(m^2^/day)/m
RIV_41	River Conductance (Ismailia Canal)	0.01	0.016	(m^2^/day)/m
RIV_42	River Conductance (Ismailia Canal)	0.01	0.022	(m^2^/day)/m
DRN_50	Drain conductance (Belbais Drain)	0.01	0.70	(m^2^/day)/m
DRN_51	Drain conductance (Belbais Drain)	0.01	0.50	(m^2^/day)/m
DRN_52	Drain conductance (Belbais Drain)	0.01	0.49	(m^2^/day)/m

**Figure 13:**
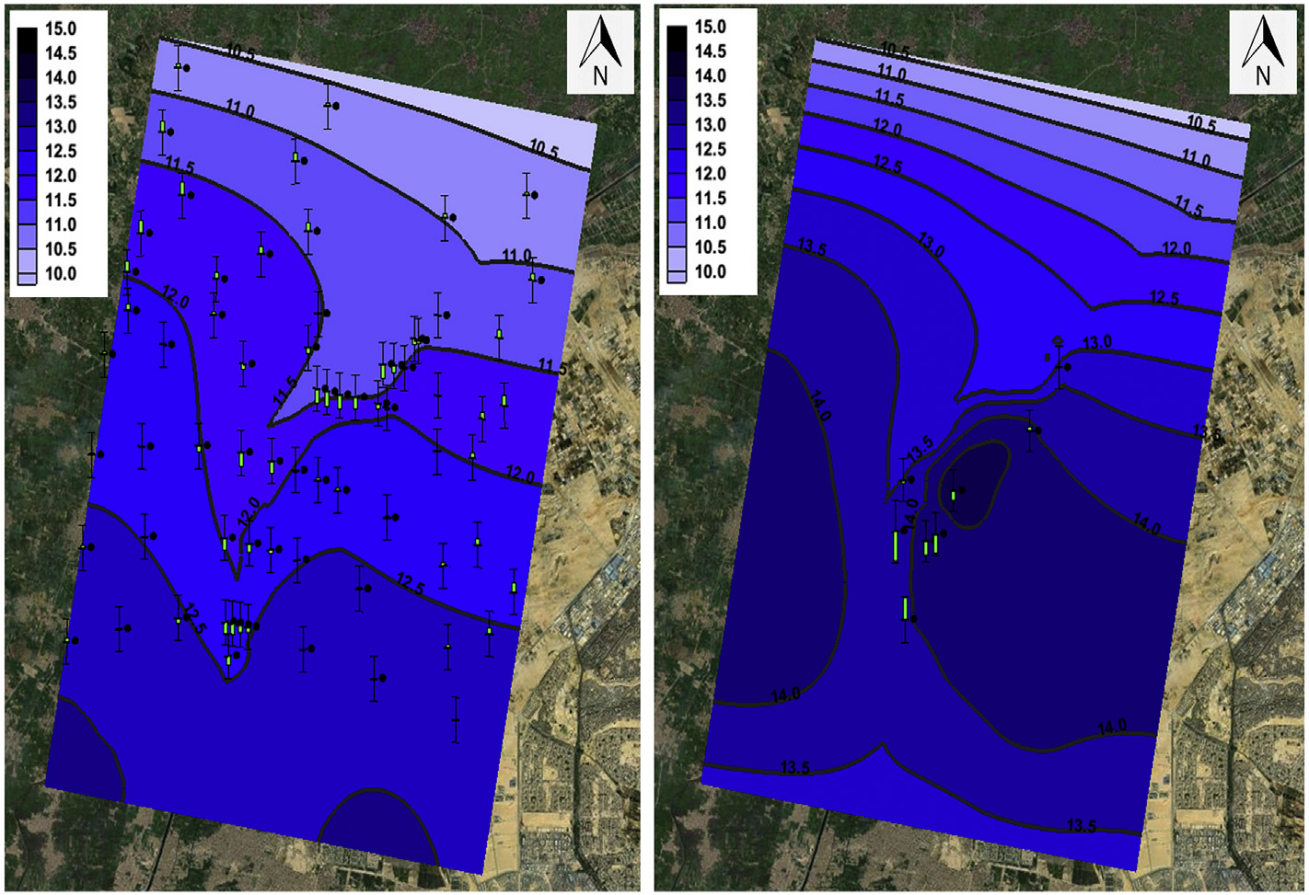
Calibrated groundwater levels for Steady-state phase (left) and Transient phase (right).

The steady-state calibration of the water levels was easier to compute, whereas, the transient phase where the parameters change with time was much more difficult due to the heterogeneity of the data. Figure 13 shows the calibrated groundwater levels for the steady-state period (1980–1990) which is based on historical groundwater level contours from the Hydrogeological map of Egypt-Nile Delta and the transient period (1990–2020) which is based on the groundwater level measurements taken during site investigation data.

**Figure 14:**
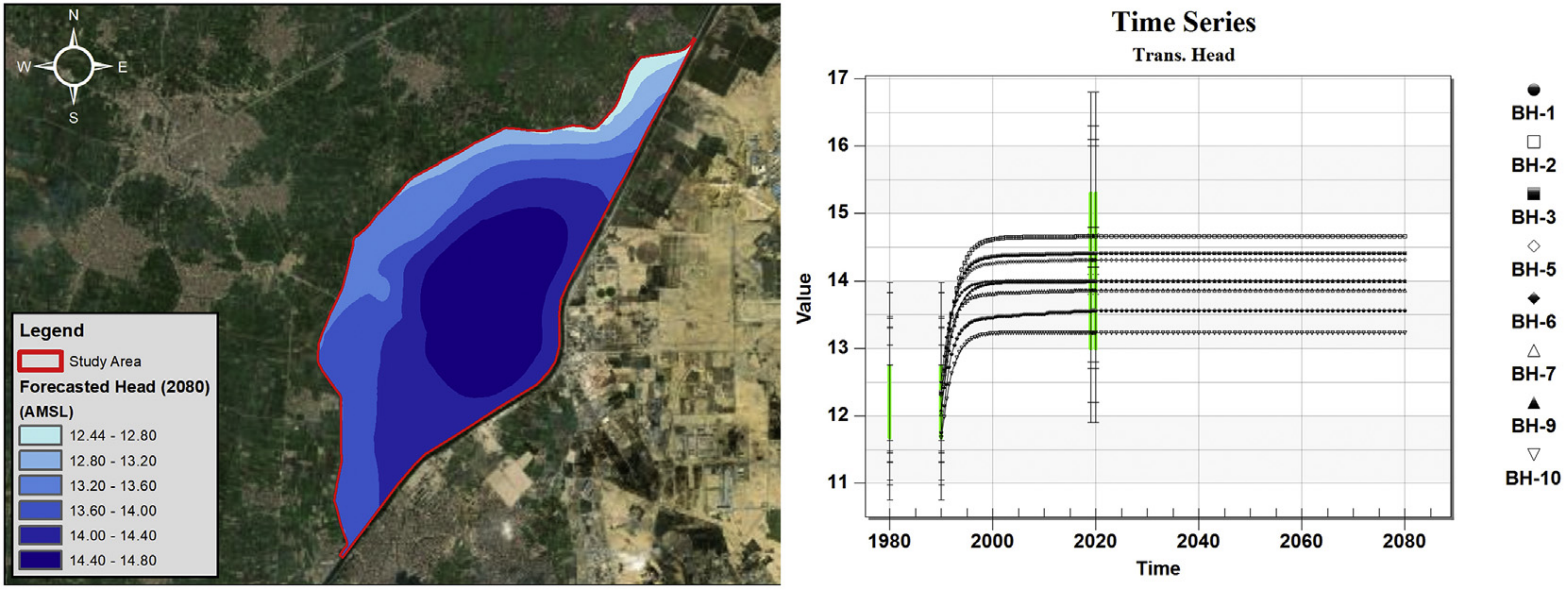
Asymptotic projected groundwater level within study area.

The estimated parameters by the calibration process are used in order to forecast the groundwater level within the study area. The groundwater level has been projected to the year 2080 to which has been chosen as the time frame limit for our study. The groundwater level has reached asymptotic behavior (i.e. steady-state behavior). This was also confirmed from the Time Series of the readings in the boreholes (calibration points) as shown in Figure 14.

### 3.2 Water quality analysis results

The results of the laboratory analysis for different parameters are recorded in Table 5. The results showed that TDS is very high within the pond and in the groundwater surrounding it. It decreases relatively as we move towards the south as indicated in the results. Boron concentrations are high at BH-05 and BH-06, while it is significantly low in the locations far from landfill (BH-06) which seems to be less affected by the boron transport. Heavy metals such as lead is also high in some locations. The level of ammonium in the Ismailia Canal upstream is 3.5 mgNH_4_/l and decreases to 1.4 mgNH_4_/l downstream. This could be due to the dumping of sewage from industries or leakage of manure and fertilizers from agricultural activities. The Biological Oxygen Demand (BOD) in Belbais Drain ranged from 24 mg/l to 30 mg/l which was a relatively low concentration taking into account that the drain usually receives huge quantities of organic waste and biodegradable materials.

**Table 5:** Groundwater and surface water samples laboratory analysis.

Location	TDS mg/l	EC (mS/cm)	pH	Bicarbonates mEq/l	Chloride mEq/l	Sulphate mEq/l	Calcium mEq/l	Magnesium mEq/l	Sodium mEq/l	Potassium mEq/l	Ammonium (mg NH_4_/l)	Nitrate (mg NO_3_/l)	Boron mg/l
IC1	288	0.45	7.9	1.42	1.02	2.53	2.25	1.64	0.96	0.11	3.50	1.4	0.286
IC2	313	0.49	7.8	1.42	1.02	2.46	2.25	1.64	0.89	0.11	1.40	1.4	0.276
Pond	14,024	17.53	7.8	2.83	89.83	74.66	23.38	29.09	114.00	0.85	1.40	2.1	0.591
BD1	844	1.32	7.2	2.17	3.56	7.10	4.51	3.29	4.61	0.43	3.50	2.8	0.402
BD2	737	1.31	7.2	2.17	4.07	6.49	5.63	2.16	4.52	0.41	3.50	7	0.393
BH-1	2,752	4.30	7.0	4.43	8.64	25.04	7.61	6.68	23.48	0.36	1.75	0.35	0.92
BH-5	9,656	12.07	7.3	3.58	56.95	62.19	15.77	22.41	83.84	0.70	1.75	0.35	2.289
BH-6	9,736	12.17	5.5	3.87	58.81	64.86	18.45	24.45	83.84	0.71	6.30	DL	2.244
BH-10	1,074	1.68	7.4	2.41	2.71	11.98	7.32	5.27	3.75	0.75	2.45	1.05	bdl
Arithmetic Mean	4,380	5.70	7.23	2.70	25.18	28.59	9.69	10.74	35.54	0.49	2.84	1.83	0.82
Maximum	14,024	17.53	7.90	4.43	89.83	74.66	23.38	29.09	114.00	0.85	6.30	7.00	2.29
Minimum	288	0.45	5.50	1.42	1.02	2.46	2.25	1.64	0.89	0.11	1.40	-	-
**Location**	**Copper mg/l**	**Iron mg/l**	**Manganese mg/l**	**Phosphorus mgP/l**	**Zinc mg/l**	**Cadmium mg/l**	**Lead mg/l**	**Aluminum mg/l**	**BOD mg/l**	**COD mg/l**	**Total Coliform cells/ml**	**Fecal Coliform cells/ml**	
IC1	0.314	0.479	0.341	0.297	0.256	0.071	0.065	0.086	-	12	-	-	
IC2	0.313	0.356	0.33	0.305	0.267	0.072	0.064	0.098	-	5.11	-	-	
Pond	0.31	0.491	0.582	0.366	0.257	0.072	0.062	0.082	10.5	238	-	-	
BD1	0.314	0.759	0.434	1.254	0.485	0.09	0.074	0.057	24.4	103	-	-	
BD2	0.36	0.865	0.432	2.12	0.29	0.073	0.065	0.065	30.5	98.4	25	13	
BH-1	bdl	2.76	1.67	0.189	bdl	bdl	bdl	0.036	-	47	-	-	
BH-5	bdl	2.126	2.587	0.155	bdl	bdl	bdl	bdl	-	47	-	-	
BH-6	bdl	21.77	3.172	0.237	bdl	bdl	bdl	bdl	-		-	-	
BH-10	bdl	5.92	0.023	0.161	bdl	bdl	bdl	0.093	-	2.62	-	-	
Arithmetic Mean	0.18	3.95	1.06	0.56	0.17	0.04	0.04	0.06	21.80	69.14	25	13	
Maximum	0.36	21.77	3.17	2.12	0.49	0.09	0.07	0.10	30.50	238	25	13	
Minimum	-	0.36	0.02	0.16	-	-	-	-	10.50	2.62	25	13	

Abbreviatations: IC: Ismailia Canal, BD: Belbais Drain, BH: Borehole, bdl: below detection limit.

For more details, see Figure 9.

### 3.3 Contaminant transport model

The groundwater mass transport model was generated using MT3DMS engine in GMS software. The results include the initial concentration levels for each of the chosen six parameters to be simulated. Additionally, the results for both scenarios of operation of the dumpsite and its closure are also represented. It is important to note that the model was built mainly to study Abu Zaabal dumpsite impact on the nearby water resources focusing mainly on the groundwater without taking into consideration any other contamination source within the study area.

**Figure 15:**
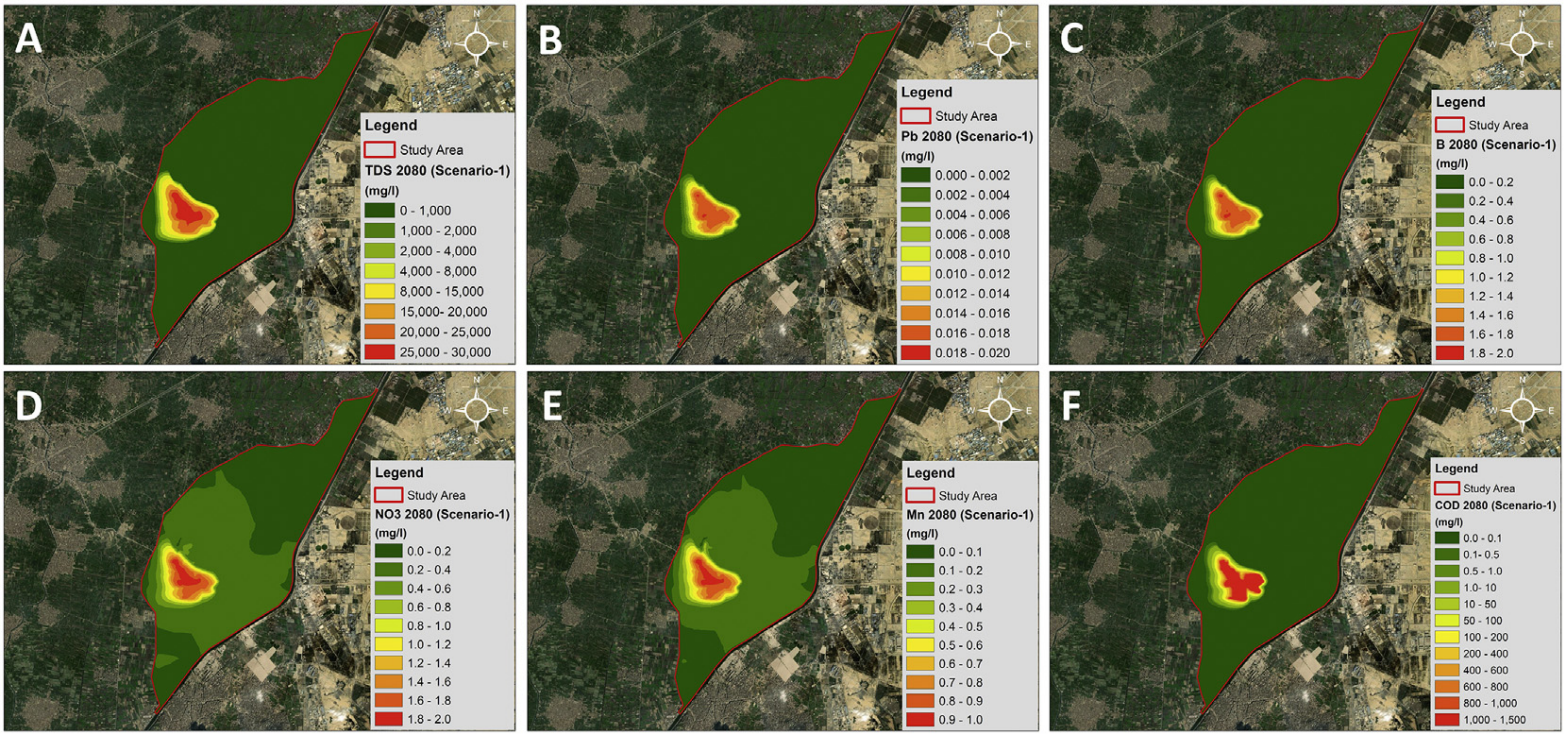
A) TDS; B) Lead; C) Boron; D) Nitrate; E) Manganese; F) COD; concentration maps for Scenario-1 2080.

For scenario-1, it is based on the operation of the dumpsite from 1980 to 2080 (i.e. 100 years). The main objective is to study the effect of the leachate generated from the dumpsite on the nearby water bodies, focusing mainly on the groundwater which is the most vulnerable along with Abu Zaabal ponds. Figure 15 shows concertation map distribution for different contaminants in year 2080.

The plume mainly moves with the flow direction and by 2080 reaches Belbais Drain and reaches a maximum concentration of 30,000 mg/l for TDS. Since the ponds are the nearest to the dumpsite (i.e. the source of contamination) they are the most affected. As the distance increases, the plume spreads using advection and dispersion, and hence the concentration decreases. Similarly, for Lead, Boron, Manganese and Nitrate, the maximum concentration values are 0.02 mg/l, 2 mg/l, 1 mg/l and 2 mg/l respectively.

COD concentration was measured during the site investigation performed in 2019 and the values ranged from 2.62 mg/l to 238 mg/l within the study area. Based on the readings, COD highest values were recorded at the ponds and Belbais Drain from the surface water samples taken. This is due to the high organic matter in wastewater dumped in the drain and the ponds. COD was the only contaminant to have the chemical reaction package used within the model. The kinetic rate of reaction (biodegradation) was chosen to be First-Order.

For Scenario-2, the scenario is based on the closing of the dumpsite (i.e. the dumpsite no longer receives any type of waste). The closure year in the model was chosen to be 2020; which is 40 years after starting the operation in 1980. The simulation was modified in order to take into account such change by reducing the recharge concentration of leachate after 2020 as the quantity of waste will no longer increase. However, the remaining solid waste which was present will still affect and will be subject to all processes such as decomposition and leachate generation will continue to form and percolate to reach the subsurface.

**Figure 16:**
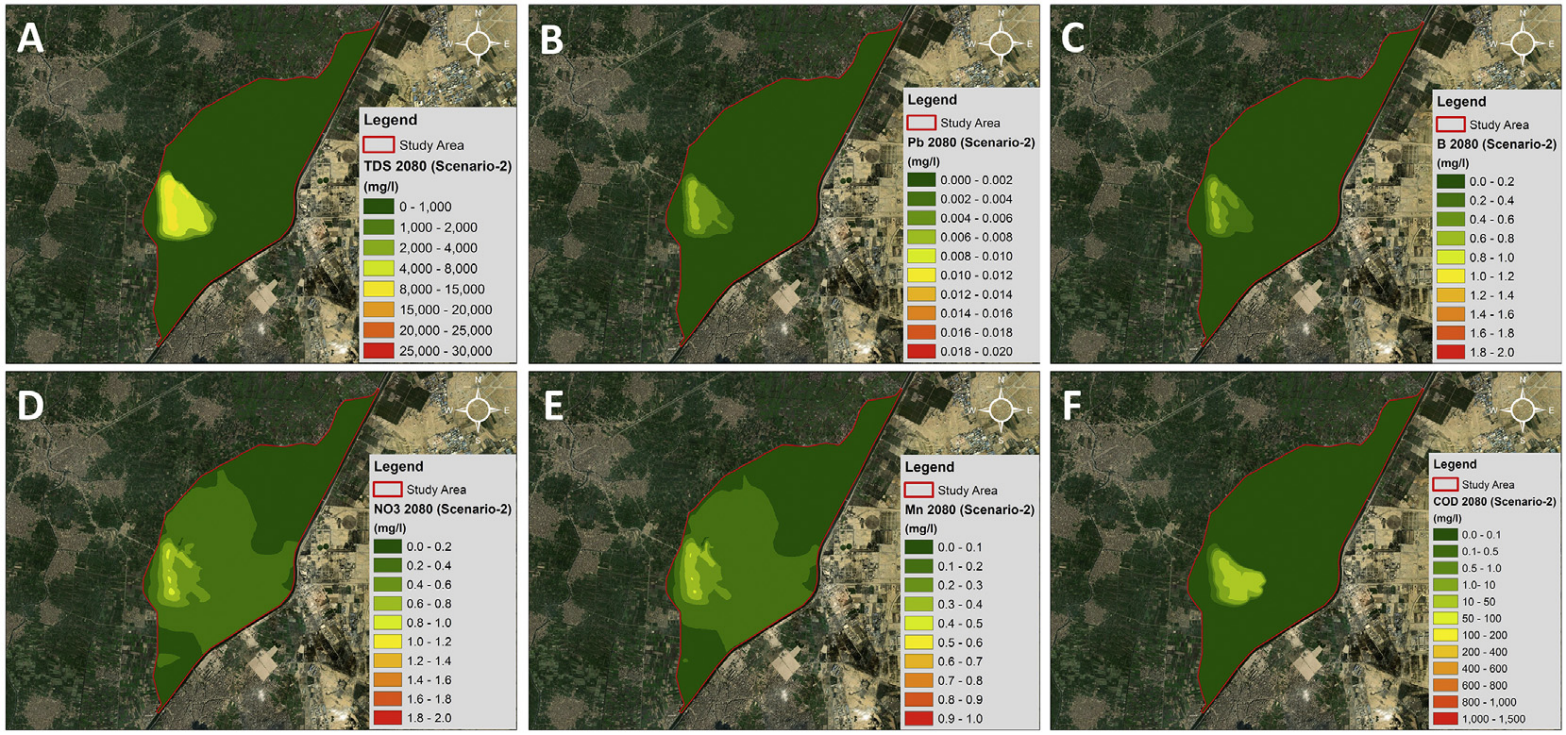
A) TDS; B) Lead; C) Boron; D) Nitrate; E) Manganese; F) COD; concentration maps for Scenario-2 2080.

The maximum concentration of the TDS in this scenario is 11,000 mg/l as shown in Figure 16. This shows a maximum reduction percentage of 60 % due to the closure of the dumpsite compared to Scenario-1. The size and extent of the plume have also changed; the contaminant concentration reaching the drain is of lower value. Similarly, for Lead, Boron, Manganese and Nitrate the maximum concentration are 0.007 mg/, 0.68 mg/l, 0.4 mg/l and 0.81 mg/l respectively. The maximum reduction percentage ranges from 60%-63% for all contaminants.

However, in the case of COD, where the biodegradation factor was included, the maximum concentration of the COD in this scenario is 25 mg/l. This shows a maximum reduction percentage of 98 % due to the closure of the dumpsite compared to Scenario-1.

### 3.4 Water quality standards comparison

In order to be able to decide on the suitability of the water to be used for different purposes such as domestic usage (i.e. drinking, bathing, etc.), agriculture or irrigation, recreational, etc.; the most important step is to compare its quality with the acceptable limits for each of the applications. Since the study region, Abu Zaabal falls in a location where mainly agriculture/irrigation and drinking water are dominant, the water quality will be compared with several international standards from different sources. These sources are the Environmental Protection Agency (EPA) [28], World Health Organization (WHO) and Initiative for Responsible Mining Assurance (IRMA) [29]. Table 6 shows the range values (minimum-maximum) of concentration from the water quality samples tested in 2019–2020 during the site investigation and the model results for Scenario-1 and Scenario-2.

**Table 6:** Comparison of site investigation and model results with Water Quality Standards [All units are in mg/l].

Contaminant	Current Value Range within study area, Site Investigation 2019	Model Results at 2020	Model Results at 2080 (Scenatio-1)	Model Results at 2080 (Scenatio-2)	Allowable Limits for Irrigation [IRMA, 2018]	Allowable Limits for Drinking [IRMA, 2018]	Allowable Limits for Drinking [EPA, 2008]
TDS	300-14,000	0-18,000	0-28,000	0-11,000	500–3500	500	500
COD	2.62–238	0–1500	0–1500	0–25	-	-	-
Nitrate	0.0001–5	0.0–1.50	0.0–2.0	0.0–0.81	-	45	10∗∗
Lead	0.0001–0.07	0.0–0.012	0.0–0.019	0.0–0.007	0.2	0.01	0.015
Boron	0.00003–2.29	0.0–1.2	0.0–1.9	0.0–0.68	0.75	2.4∗	2.4∗
Manganese	0.023–3.17	0.0–0.7	0.0–1.0	0.0–0.4	0.2	0.05	0.05

**Notes:** - (No Information).

∗ WHO (World Health Organization).

∗∗ (Nitrate measured as Nitrogen).

The results show that there is a deterioration in the water quality of the groundwater and surface water within the study area. Based on the comparison shown in Table 6, the water cannot be used for domestic (drinking) purpose as the current concentration within the site and forecasted values using the model exceeds the allowable limits. On the other hand, for Irrigation purposes, the water quality is not suitable for most of the contaminants.

The results of the mass transport model also showed that not all the contamination is coming from the dumpsite and there are other sources of contamination as stated before which negatively affects the surrounding water resources. As a result, the following recommendations are proposed:

i. Continuous detailed study to be performed by taking periodic samples from groundwater and adjacent waterways (Belbais Drain, Ismailia Canal and Abu Zaabal ponds) to determine the sensitivity of the effect of migration of pollutants.
ii. As shown from the second scenario performed which simulated the closure of the dumpsite, which has proven to decrease the contamination in the future. Therefore, the dumpsite needs to be closed to not receive any further waste.

Following the above recommendations which are based on the site investigation, model results and analysis performed, it is also important to highlight that the dumpsite also is a great threat in terms of aesthetics. This is due to the view and the foul odor coming from it. Moreover, huge quantities of methane gas are produced which causes a lot of fire and air pollution to the nearby urbanized area which was confirmed during the site inspection. Hence, the dumpsite should be shut down as soon as possible.

The comprehensive research carried out was beneficial in order to clarify the impacts Abu Zaabal dumpsite had on the surrounding region. However, there was a few limitations which was faced that affected the completeness of the study. The difficult site conditions prevented certain boreholes to be drilled and water samples to be taken from deep depths within the dumpsite (leachate). There was also lack of data and previous studies within the study area. No pumping tests data were available for the aquifer in order to estimate the hydraulic properties. The location of the study area was very sensitive in terms of people culture and working environment.

There are a number of gaps in our knowledge in research that follow from our findings, and would benefit from further research, including realist evaluation to extend and further test the theory we have developed here. For future similar research studies, it is important to take into consideration other factors which may contribute to the contamination of an aquifer. In this study, the dumpsite was the focal point as a contamination source, but in reality, there are other elements which may lead to groundwater contamination especially in developing countries. The mass transport model could be enhanced by having several water level readings through the years and during different seasons. This should also be done for the water quality analysis. Finally, since the MT3DMS engine in GMS does not support calibration using PEST or any other module, it is recommended to perform the calibration process for the mass transport model using another method. This will increase the reliability and accuracy of the model.

## 4 Conclusion

In this paper, a case study for a solid waste dumpsite located in Abu Zaabal, South-East of the Nile Delta, Qalyubiyah Governorate, Egypt was presented where the dumpsite receives a huge quantity of waste. The paper can be considered as an attempt to develop a methodology for studying similar cases around the world and highlight the hazards from indiscriminate dumping of solid wastes which is still followed in most of the in the developing countries. The main objective was to study the effect of the leachate generated from the solid waste at Abu Zaabal dumpsite on the nearby water resources. Hence, a combined groundwater flow and mass transport model were constructed using historical data from literature and new data obtained from the site investigation performed in 2019. The site investigation consisted of several fieldwork tests that was crucial in the study and preparing the model. The Topographic survey performed using GPS showed that the ground elevation variations (14–25 AMSL) within the site and that Abu Zaabal ponds fall within a zone of depression at 11.85 AMSL. Eight (8) boreholes were drilled at depths ranging from 10m to 25m in order to study the lithological formations which forms the shallow aquifer. The main soil formations encountered were sand, sandstone and basalt. Groundwater depths were measured within each of these boreholes as well as the surface water levels at Abu Zaabal ponds, and, upstream and downstream of Ismailia Canal and Belbais Drain. The groundwater depth within the site was between 2.8m to 5.5m. Water samples were taken from selected locations at the boreholes, ponds, canal and drain. The results of the water quality parametric tests performed on these samples indicated that the concentration of TDS is very high. The heavy metals such as lead was also relatively high especially within Belbais Drain. Other elements and ions were also tested and the overall conclusion was that the water was not suitable for any usage without treatment.

From this model, it was able to analyze the effect of the dumpsite on the water quality of the surrounding water bodies. The groundwater model was calibrated, and the projected results showed that there is an increase in the groundwater table by 2–3 m between 1980 and 2019. The ponds also lie in a relatively low permeable zone where consists mainly of fractured basalt rocks and sandstone. The mass transport model was simulated from 1980 until 2080 mainly to study the effect in the long run based on two different scenarios.

The results indicated that the leachate from the dumpsite mainly affects the water quality of the groundwater, ponds and the nearby Belbais Drain by a radius of approximately 3 Kilometers. On the other hand, the nearby freshwater Ismailia Canal being upstream the flow direction is not affected by the dumpsite. The presence of the basalt rocks within the region of the dumpsite acted as a natural barrier and hindered the contamination and limited its flow extent. The simulated model showed that the dumpsite generates a huge number of contaminants due to the leachate formation. The two scenarios developed indicated that the dumpsite closure is crucial as the concentration of the contaminants decreased by a range of 60–65%. The water quality parameters tested for different samples from the site indicated that there is a current deterioration in the groundwater and surface water quality surrounding the dumpsite.

## Declarations

### Author contribution statement

Mohamed E. El-Mathana: Conceived and designed the experiments; Performed the experiments; Analyzed and interpreted the data; Contributed reagents, materials, analysis tools or data; Wrote the paper.

Nagwan G. Mostafa: Analyzed and interpreted the data; Wrote the paper.

Mona M. Galal: Contributed reagents, materials, analysis tools or data; Wrote the paper.

Abdelsalam Elawwad: Conceived and designed the experiments; Performed the experiments; Contributed reagents, materials, analysis tools or data; Wrote the paper.

### Funding statement

This work was supported by 10.13039/501100002377Cairo University, Faculty of Engineering.

### Data availability statement

Data included in article/supplementary material/referenced in article.

### Declaration of interests statement

The authors declare no conflict of interest.

### Additional information

No additional information is available for this paper.
